# Successful treatment of refractory subacute cutaneous lupus erythematosus with deucravacitinib

**DOI:** 10.1016/j.jdcr.2023.07.030

**Published:** 2023-08-04

**Authors:** Nicole Bouché, Miriam A. Al-Saedy, Eingun J. Song

**Affiliations:** a Elson S. Floyd College of Medicine, Spokane, Washington; b Frontier Dermatology Partners, Mill Creek, Washington

**Keywords:** biologics, clinical research, drug response, immunodermatology, general dermatology, medical dermatology

## Introduction

Cutaneous lupus erythematosus (CLE) is an autoimmune disorder involving a broad range of dermatologic manifestations that can occur in the presence or absence of associated systemic lupus erythematosus (SLE).^1^ Subacute cutaneous lupus erythematosus (SCLE) is one of 3 main subtypes of CLE that are differentiated according to histopathology and clinical findings and can be triggered by endogenous or exogenous factors.^2^ Treatment for SCLE traditionally involved a combination of preventive strategies, topical therapies (such as corticosteroids or calcineurin inhibitors), and systemic therapies for widespread disease, including oral antimalarials, systemic corticosteroids, immunosuppressants, and biologic therapies.^1^ Although there have been significant advancements in understanding the immunopathogenesis of lupus erythematosus, there is a lack in development of more targeted immune-related therapies, and SCLE remains a difficult disease to treat.^3^

Deucravacitinib is an oral, selective, allosteric tyrosine kinase 2 (TYK2) inhibitor approved for the treatment of adults with moderate-to-severe plaque psoriasis, who are candidates for systemic therapy or phototherapy.^4^ Deucravacitinib previously demonstrated efficacy in a phase II trial, with an acceptable tolerability in reducing SLE disease activity, thus supporting the potential benefits of TYK2 inhibition in systemic diseases.^5^ Additionally, there is a phase II study that is currently investigating the efficacy of deucravacitinib in participants with moderate-to-severe discoid lupus erythematosus and/or SCLE, regardless of whether the systemic disease is well controlled with the current standards of care therapy.^6^ Herein, we report a case of treatment refractory SCLE that was successfully treated with deucravacitinib therapy.

## Case report

**Fig 1:**
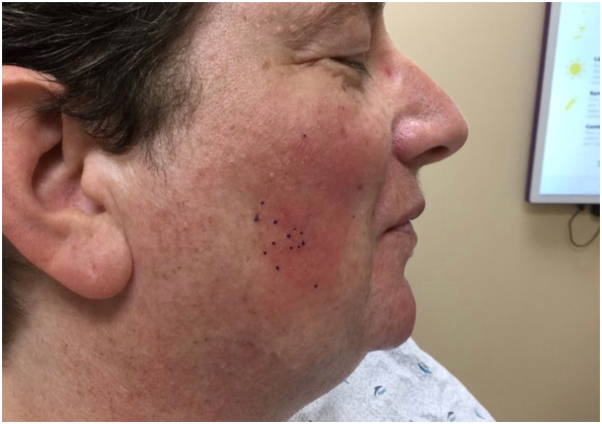
Initial presentation before the treatment.

A 51-year-old Caucasian woman with no personal or family history of atopy, psoriasis, or autoimmune disease presented for a second opinion of a persistent, mildly pruritic, red, scaly rash on her right cheek. The rash first appeared 1 year before the presentation and gradually worsened. She was previously treated with low potency topical corticosteroids, topical azoles, metronidazole, and doxycycline. On examination, she had an erythematous scaly thin plaque confined to the right malar cheek (Fig 1). The rest of her skin exam was unremarkable, including her scalp, retroauricular and nasolabial folds, elbows, knees, and nails. She was initially treated with desonide 0.05% cream and tacrolimus 0.1% ointment with just minimal improvement. Expanded series patch testing did not reveal any relevant contact allergens. A 4-mm punch biopsy was performed on the right cheek, which showed a superficial-to-deep perivascular lymphocytic infiltrate with rare eosinophils, focal interface changes, thickened basement membrane, and moderately increased stromal mucin. Based on the clinical presentation, histopathology, and the lack of response to treatments for other entities such as rosacea, seborrheic dermatitis, atopic dermatitis, and psoriasis, the patient’s presentation was the most consistent with SCLE.

Review of systems was notable for the rash worsening in the sun but otherwise unremarkable for any symptoms of SLE. Laboratory evaluation was unremarkable, including normal results for CBC, CMP, ANA, SSA/SSB, and dsDNA. The patient’s medication history included topical estrogen cream, lisinopril, and hydrochlorothiazide. Owing to the concern of possible drug-induced SCLE, hydrochlorothiazide was stopped.

**Fig 2:**
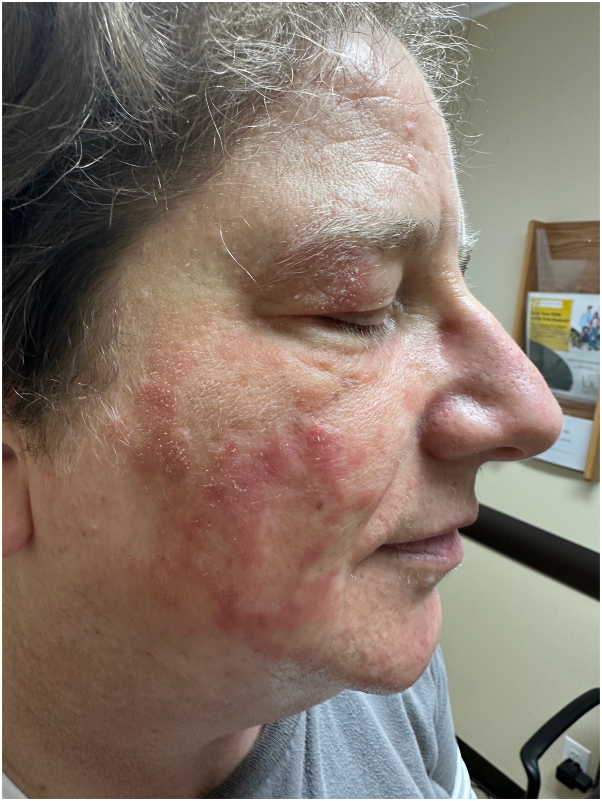
During the treatment with mycophenolate mofetil, before starting deucravacitinib.

**Fig 3:**
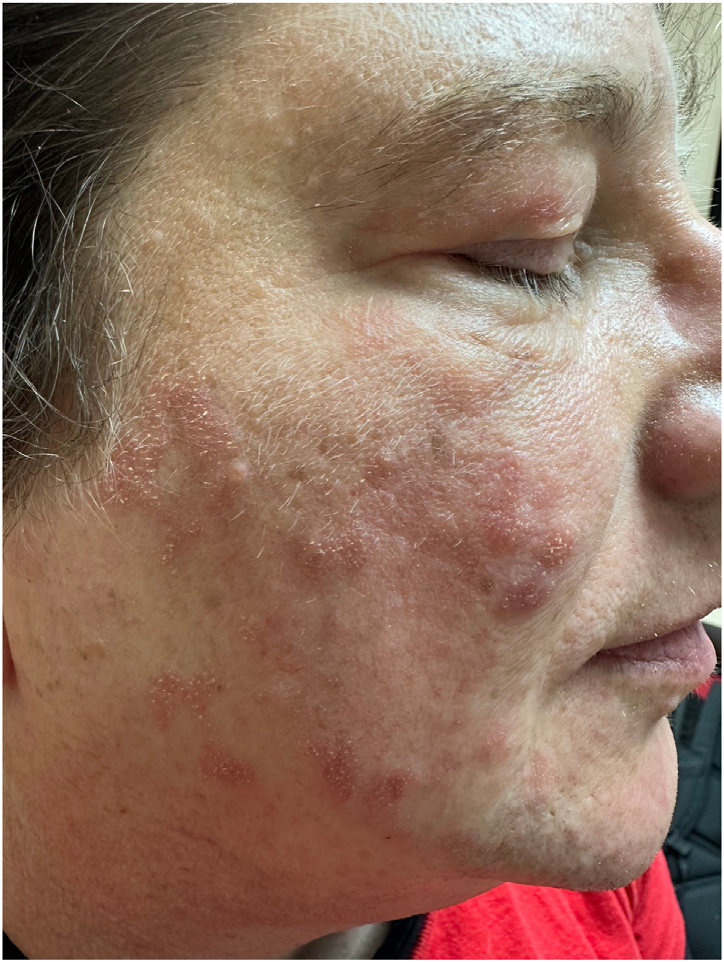
Eight weeks after the treatment with deucravacitinib (6 mg daily).

**Fig 4:**
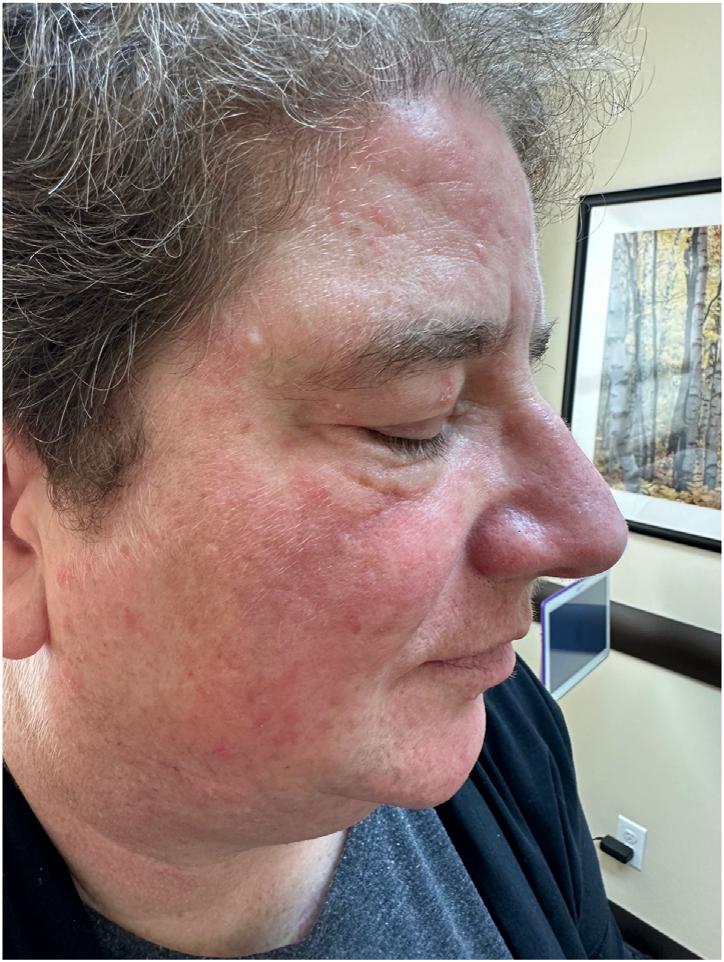
Four months after the treatment with deucravacitinib (6 mg daily).

Patient was subsequently started on hydroxychloroquine (maximum dose of 200 mg twice daily) for a total of 6 months, then methotrexate (maximum dose of 22.5 mg per week), and finally mycophenolate mofetil (maximum dose of 3 gm daily), while alternating with ultrapotent topical corticosteroids and tacrolimus ointment. However, the patient failed to show any appreciable improvement (Fig 2). Repeat biopsy was performed, which showed similar findings to the initial biopsy conducted 2 years earlier. Decision was made to discontinue all prior immunosuppressants and try off-label use of deucravacitinib monotherapy at the dose of 6 mg daily. At her 8-week follow-up, the condition was at least 50% improved (Fig 3). By her 4-month follow-up, the condition was nearly completely clear (Fig 4), and the patient continued to tolerate the medication well. The patient is currently in the process of applying for foundational support from the drug manufacturer.

## Discussion

In this case report, we see a potential benefit of deucravacitinib in the treatment of SCLE. The TYK2 pathway is responsible for mediating immune signaling of cytokines involved in the pathogenesis of SLE, including type I interferons (IFN), interleukin-10, interleukin-12, and interleukin-23.^7^ It has been found that type I IFN signaling and expression of type I IFN proteins are also upregulated in the lesional skin of patients with CLE, indicating a potentially shared mechanism and common proinflammatory markers.^8^ While all the Janus kinases (JAKs) play a critical role in various immune responses, JAK1 to 3 are involved in facilitating a series of signals that support the larger systemic processes, including hematopoiesis, bone regulation, and lipid metabolism.^7^ By uniquely binding to the TYK2 regulatory domain, deucravacitinib is substantially more selective than other JAK inhibitors with little to no activity against JAK1 to 3, indicating that it can inhibit downstream cytokine signaling without interfering with other critical systemic functions.

The main limitation of this study is that this is a single case report. Although there are many management strategies available for SCLE, the degrees of efficacy are varied, and resistance to conventional treatments is common.^9^ Further studies investigating the safety, efficacy, and tolerability of deucravacitinib for the treatment of patients with SCLE are necessary. Assessment of longer-term response, maintenance of clinical response after drug cessation, and comparison studies with biologic agents will be the next important steps to understand the role of deucravacitinib in the treatment landscape for SCLE.

## Conflicts of interest

Eingun James Song is paid consultant at BMS, AbbVie, Eli Lilly, Janssen, Novartis, UCB, Pfizer, Amgen, Dermavant, Arcutis, Incyte, SUN, Boehringer Ingelheim, Sanofi, and Regeneron. Nicole Bouché and Miriam A. Al-Saedy have no conflicts of interest to declare.
